# Time-Lapse Embryo culture: A better understanding of embryo development and clinical application

**DOI:** 10.5935/1518-0557.20210107

**Published:** 2022

**Authors:** Silvana Márquez-Hinojosa, Luis Noriega-Hoces, Luis Guzmán

**Affiliations:** 1 PRANOR Laboratorio. Grupo de Reproducción Asistida, Lima, Peru; 2 Clínica Concebir. Calle Los Olivos 364, San Isidro, Lima, Peru

**Keywords:** blastocyst, aneuploidy, embryo culture

## Abstract

Conventional embryo assessment is performed by removing embryos from incubators at least once a day. However, it is static and limited to specific time points, reducing the amount of information that could potentially be obtained. Fortunately, the time-lapse system is a powerful technology that enables to observe embryo development progression by image acquisition at recurrent time intervals, without interfering in the culture conditions. There are numerous studies that used time-lapse incubators, focusing on embryo kinetics, patient characteristics and clinical outcomes. This review aims to find agreements in the literature concerning embryo kinetics and patient characteristics: age, body mass index, smoking habit, polycystic ovary syndrome and endometriosis; as well as culture conditions that involved culture media and oxygen concentration. Furthermore, they showed differences according to ploidy status, direct/reverse cleavage, gender and the potential association between embryo collapse and clinical outcomes.

## INTRODUCTION

Since the beginning of assisted reproduction techniques (ART), morphology has been used as a standard method for embryo selection, generating comparable clinical outcomes among laboratories (Ludwig *et al*., 2000). Currently, several laboratories are culturing embryos until the blastocyst stage, and the best morphology-embryo is selected for embryo transfer (Scott *et al*., 2000).

The Time-Lapse (TL) system in ART was first described by Payne *et al*. (1997), who reported morphokinetic events in early embryo development stages, some of them were: second polar body extrusion, pronuclear formation (maternal and paternal) and cellular cleavage. Morphokinetics enables to compare time frames and dysmorphic events during embryo development (Payne *et al*., 1997). With all this information, algorithms have been proposed with the aim to increase implantation rates in ART (Sakkas *et al*., 1998; Gardner *et al*., 2015).

Embryo culture in TL incubators enabled us to continuously monitor embryo development, without interfering with culture conditions (temperature, gas concentrations and pH); thus, decreasing the negative impact on embryo development (Kahraman *et al*., 2020) and minimizing the negative effect of standard embryo evaluation under conventional microscopes.

The clinical effectiveness of TL systems is still controversial. A meta-analysis concluded that the TL was associated with a significantly higher ongoing pregnancy, live birth rate and significantly lower early pregnancy loss in comparison to the conventional incubator and morphological selection (Pribenszky *et al*., 2017); whereas a recent Cochrane review concluded that there are insufficient evidence to support a higher live birth rate when the TL system is used with the aid of software assessment (Armstrong *et al*., 2018). Furthermore, similar meta-analyses have demonstrated some clinical benefits using TL (Armstrong *et al*., 2018; Chen *et al*., 2017). This study aimed to describe how morphokinetic parameters differ according to patient characteristics and culture conditions during embryo development.

## PATIENT CHARACTERISTICS AND EMBRYO MORPHOKINETICS

### Age

It is widely accepted that older women have diminished ovarian reserve and poor oocyte quality (Navot *et al*., 1991). Additionally, old patients have higher chances to produce aneuploid embryos, which reduce pregnancy rates (Grøndahl *et al*., 2017).

Retrospective studies concluded that there are no significant differences in morphokinetic parameters (Tables 1 and 2) during cleavage stages between the embryos from advanced maternal age and those of younger women (Hickman *et al*., 2013; Silva *et al*., 2015; Gryshchenko *et al*., 2014; Warshaviak *et al*., 2019). By contrast, two retrospective studies reported a significantly faster cleavage in some kinetic parameters in younger patients when compared to older ones (Akarsu *et al*., 2017; Dal Canto *et al*., 2021). Nevertheless, these differences are not evident in patients aged ≥ 37 years (Dal Canto *et al*., 2021).

**Table 1.** Definition of time-lapse morphokinetic parameters.

Morphokinetic variables	Description
tPB	Appearance of second polar body
tPNa	Appearance of the first pronuclei
tPN	Appearance of both pronuclei
tPNf	Time when both pronuclei had faded
t2	Time to 2-cell embryo
t3	Time to 3-cell embryo
t4	Time to 4-cell embryo
t5	Time to 5-cell embryo
t6	Time to 6-cell embryo
t7	Time to 7-cell embryo
t8	Time to 8-cell embryo
t9	Time to 9-cell embryo
tSC	Time from insemination to initiation of compaction
tM	Time from insemination to formation of a morula, completion of compaction process
tSB	Time from insemination to star blastulation
tB	Time form insemination to formation of a full blastocyst
tEB	Time to expanded blastocyst
tH	Time to hatching blastocyst, the trophectoderm herniation through the zona pellucida
tHatched	Time to hatched blastocyst, the embryo has full escaped the zona pellucida
**Duration variables**	**Description**
cc1	Time of first cell cycle (t2- tPB)
cc2	Time of second cell cycle (t3-t2), from 2 to 3 cells
cc3	Time of third cell cycle (t5-t3), from 3 to 5 cells
s2	Time of synchrony of the second cycle (t4-t3), from 2 to t4 cells
s3	Time of synchrony of the third cell cycle (t8-t5), from t5 to 8 cells.

**Table 2.** Summary of the studies highlighting the morphokinetic variables with statistically significant differences.

Item	Reference	Type of study	Embryos (n)	Patients (n)	Embryo culture	Day transfer	Morphokinetic parameters		Statistically significant variables
							significant difference	no significant difference	
Young vs Older	Hickman *et al*., 2013	Retrospective	348	NA	Blastocyst stage	NA	x		tM, tEB,tHed
	Silva *et al*., 2015	Retrospective	288	76	Blastocyst stage	NA	x		tM,tSB,tB,tEB
	Akarsu *et al*., 2017	Retrospective	1144	197	Cleavage stage	d3	X		tPNf,t2,t3,t4
	Dal Canto *et al*., 2021	Retrospective	4915	1066	Cleavage stage	d2/d3	X		tPNf,t2,t3,t4 ,t8
	Gryshchenko *et al*., 2014	Prospective	262	616	Cleavage/Blastocyst stage	d3/d5		X	
	Warshaviak *et al*., 2019	Retrospective	1148	122	Cleavage stage	d3		X	
BMI	Bartolacci *et al*., 2019	Retrospective	7180	1528	Cleavage/Blastocyst stage	d3/d5	X		t5,t8
	Bellver *et al*., 2013	Retrospective	424	89	Blastocyst stage	d3	X		t2,t3,t4 and t5
	Lammers *et al*., 2013	Retrospective	NA	366	Cleavage stage	NA	X		t4,t5,t8
	Leary *et al*., 2015	Retrospective	155	29	Blastocyst stage	d3/d5	X		tM
	Mumusoglu *et al*., 2017	Retrospective	415	103	Blastocyst stage	NA	X		tPN,tPNf,t2,t4,t5,t6,t7,t8,s2
	Silva *et al*., 2015	Retrospective	288	76	Blastocyst stage	NA		X	
PCOS vs non PCOS	Chappel *et al*., 2017	Retrospective	1108	86	Blastocyst stage	NA	X		t7,t8,t9,tM
	Chappell *et al*., 2020	Retrospective	1618	256	Blastocyst stage	NA	X		t5,t6,t7,t8,t9, tSC
	Sundvall *et al*., 2015	Prospective	1388	217	Blastocyst stage	d6	X		tSC, tM and t5-t4
	Tabibnejad *et al*., 2019	Prospective	547	100	Cleavage stage	d3	X		tPNf,t2,t3,t4,t5,t6,t7,t8
	Tam Le *et al*., 2019	Prospective	851	106	Cleavage stage	d2		X	
	Wissing *et al*., 2014	Prospective	347	71	Blastocyst stage	d2	X		tPNf,t2,t3,t4,t7
Endometriosis	Boynukalin *et al*., 2019	Retrospective	439	NA	Cleavage stage	NA	X		tPB,tPN,cc1,s2
	Freis *et al*., 2018	Retrospective	477	168	Blastocyst stage	NA	X		cs2-8 = ((t3-t2)+(t5-t4))/(t8-t2)) cs4-8 = (t8-t5)/(t8-t4)
	Schenk *et al*., 2019	Retrospective	1148	163	NA	d3/d4/d5	X		s2, t9
Smoking habitus	Fréour *et al*., 2013	Retrospective	485	135	Cleavage/Blastocyst stage	d3/d5	X		t3,t4,t5
	Siristatidis *et al*., 2015	Prospective	NA	239	Cleavage stage	d2/d3	X		cc2, S2
Single *vs*. sequential media	Basile *et al.*, 2013	Prospective	532	75	Cleavage stage	d3		X	
	Ciray *et al*., 2012	Prospective	319	51	Blastocyst stage	d3/4/5	X		tPNf,t2,t3,t4,t5
	Costa-Borges *et al*., 2016	Prospective	628	59	Blastocyst stage	d5		X	
	Hardarson *et al*., 2015	Prospective	1356	128	Blastocyst stage	d3/5/6	X		t7,t8, cc2
VF *vs*. ICSI	Bodri *et al*., 2015	Retrospective	1285	209	Blastocyst stage	d5	X		tBE
	Cruz *et al*., 2013	Retrospective	1203	178	Blastocyst stage	d3/5		X	
	Dal Canto *et al*., 2012	Retrospective	459	NA	Blastocyst stage	d3/5	X		t2, t3
	Kim *et al*., 2017	Cohort study	1830	NA	Blastocyst stage	NA	X		tPNf,t1,t2,t3,t4,t5, t6
	Lemmen *et al*., 2008	Cohort study	102	NA	Cleavage stage	d2	X		t2
Oxygen 5% *vs*. 20%	Kirkegaard *et al*., 2013a	Retrospective	363	84	Blastocyst stage	NA	X		s3

Note: NA: not available; d2/3/5: day 2/3/5; other abbreviations in Table 1.

On the other hand, morphokinetics is faster in later stages (tM, tSB, tB and tHatched) in younger patients (<36 years) than their counterparts; it becomes slower with increasing maternal age (Hickman *et al*., 2013; Silva *et al*., 2015).

### Body Mass index (BMI)

BMI is defined as the body weight in kilograms divided by the square of height in meters; it is universally expressed in units of kg/m^2^, used to broadly categorize a person as underweight (<18,5 kg/m^2^), normal weight (18.5 to 24.5 kg/m^2^), overweigh (25 to 30 kg/m^2^) or obese (over 30 kg/m^2^) (WHO, 2020).

### Overweight and obesity

Obese women need longer time to become pregnant (Gesink Law *et al*., 2007; Wise *et al*., 2010) and their risk of infertility is almost three times higher compared to non-obese women (Zaadstra *et al*., 1993). Obese women who underwent fertility treatments have lower number of retrieved and mature oocytes, poorer embryo quality (Bellver *et al*., 2013), lower fertilization (Krizanovská *et al*., 2002) and increased miscarriage rates (Rittenberg *et al*., 2011).

Classical morphological evaluation does not seem to be useful for analyzing the impact of obesity on embryo quality (Bellver *et al*., 2010; Shah *et al*., 2011). However, morphokinetic parameters showed differences. A recent study, found that t5 and t8 were delayed in obese and in overweight women in relation to the normal-weight group (Bartolacci *et al*., 2019); while another study reported that embryos generated from obese and normal-weight infertile women showed similar cleavage patterns, but both groups were slower in early cleavage stages (t2, t3, t4 and t5) than embryos from fertile donors (control) (Bellver *et al*., 2013). In contrast, other studies reported that higher BMI (>25 kg/m^2^) was associated with earlier occurrence of tM (Leary *et al*., 2015) as well as earlier tPN, tPNf, t2, t4, t6, t7 and t8 and S2 than normal-weight women (Mumusoglu *et al*., 2017).

### Under weigh

There is no strong evidence that underweight women are associated with menstrual abnormalities or infertility. However, TL systems have enabled to study the morphokinetics of these patients. A study found that underweight women presented later t4, t5, t6 and t8 cell stages than their normal-weight, overweight and obese counterparts (Lammers *et al*., 2013). By contrast, a study reported that BMI (low or high) does not impact embryo morphokinetics (Silva *et al*., 2015).

### Polycystic Ovary Syndrome (PCOS)

PCOS affects 5-10% of women of reproductive age. It is considered a heterogeneous disorder. It is identified using the Rotterdam criteria, in which two out of three of the following conditions must be met: the presence of oligo- and/ or anovulation, clinical and/ or biochemical signs of hyperandrogenism and polycystic ovaries.

Morphokinetic parameters in hyper-androgenic PCOS women had a delay in early stages (from tPNf to t8) compared with embryos from non-PCOS regular cycles, but there was no kinetic difference between normo-androgenic PCOS and control women (Wissing *et al*., 2014). In addition, another report stated that embryos generated from PCOS patients had long developmental timings (tPNf, t2, t3, t4, t5, t6, t7 and t8) compared to normo-ovulatory women (Tabibnejad *et al*., 2019).

Nevertheless, other studies reported a faster development in PCOS and hyper-androgenic patients when compared to embryos from non-PCOS patients during the cleavage stage (t5, t6, t7, t8, t9 and 4^th^ cleavage division (from 8-cells to 9 or more cells), time to tSC and tM (Sundvall *et al*., 2015; Chappell *et al*., 2017; 2020).

On the other hand, a recent paper documented any significant differences in early embryonic development between the PCOS group and control patients. However, we must emphasize that in this study, the embryos were cultured until day 2 (Tam Le *et al*., 2019).

Finally, the current available data concluded that the implantation rate and the clinical pregnancy rate did not differ between PCOS patients and their control counterparts (Wissing *et al*., 2014; Sundvall *et al*., 2015; Tabibnejad *et al*., 2019; Tam Le *et al*., 2019). Retrospective studies concluded that PCOS patients have a trend towards a lower live birth rate and higher spontaneous abortion rates (Chappell *et al*. 2017; 2020).

### Endometriosis

Endometriosis is defined as the presence of endometrial glands and stroma cells growing outside the uterus (Burney & Giudice, 2012). Endometriosis affects 10-15% of all women in reproductive age (Giudice & Kao, 2004). It is associated with chronic pelvic pain and infertility (Parasar *et al*., 2017), and it is subdivided into four categories: stage I (minimal), stage II (mild), stage III (moderate) and stage IV (severe) (Johnson *et al*., 2017).

One study reported that endometriosis influences morphokinetic parameters without considering the severity of the disease; especially in early embryo stages (Freis *et al*., 2018). However, a deeper analysis associated with the endometriosis stages reported that patients with severe endometriosis reached t9 faster than patients with minimal or moderate endometriosis, as well as the control group (patients without endometriosis). Moreover, the synchronicity of the two blastomere divisions within the second cell cycle (s2) was faster in the endometriosis group than in women without endometriosis (Schenk *et al*., 2019).

On the other hand, a recent study concluded that embryos from endometriotic patients had different kinetics during cleavage stages, because they had a shorter cc1 and longer tPB, tPNa; as well as s2 than control group patients (Boynukalin *et al*., 2019).

### Smoking

Its negative impact on fertility has been widely described in women and men, affecting gamete quality and embryo development (Waylen *et al*., 2009). Smoker women have a higher risk of fertilization failure and lower implantation rates (Gruber *et al*., 2008). Furthermore, smoking causes obstetric complications, such as miscarriages, preterm births and premature membrane rupture (Levis *et al*., 2014).

We know that smoking affects at least one of the embryological events in the early cleavage stage of development (Siristatidis *et al*., 2015). A study found that embryos from smokers are significantly slower during cleavage stages (t3, t4 and t5), than those from nonsmokers; curiously, there are no kinetic differences between embryos from smokers and nonsmokers that stopped their development (Fréour *et al*., 2013).

## CULTURE MEDIA: SINGLE AND SEQUENTIAL MEDIA

Embryos can be cultured in uninterrupted culture using a single medium throughout the 5/6 days of culture or sequential media, where two media with different composition are used sequentially (Costa-Borges *et al*., 2016).

Studies with oocytes from donors and patients have not reported differences in morphokinetic parameters between single step media and sequential media in embryo cultures until days 3 or 5/6 (Basile *et al*., 2013; Costa-Borges *et al*., 2016).

On the other hand, a study using autologous oocytes suggested that embryos cultured in a single media have faster development from tPNf until t5 than those cultured in sequential media (Ciray *et al*., 2012); while a multicenter clinical trial reported a delay in t7, t8 in embryos cultured in single media (Hardarson *et al*., 2015).

Interestingly, besides the differences in morphokinetics, the studies did not find differences in implantation rate, ongoing pregnancy rates and live birth between the groups (Ciray *et al*., 2012; Basile *et al*., 2013; Hardarson *et al*., 2015; Costa-Borges *et al*., 2016). However, single media simplifies the logistics of an IVF laboratory, and the embryos can be assessed without disturbing culture conditions through the time-lapse system.

## FERTILIZATION TECHNIQUE

Oocytes are inseminated using conventional in vitro fertilization (IVF) or Intracytoplasmic sperm injection (ICSI). When IVF is performed, some sperm selection processes are maintained in vitro. These processes are sperm penetration of the cumulus cells, zona pellucida interaction and fusion of the oolemma, whereas during ICSI a single sperm is injected into a mature oocyte.

These two processes are different, and it is not possible to determine exactly when fertilization occurs in IVF. Some time-lapse studies reported a faster development in embryos generated by ICSI than in IVF (Lemmen *et al*., 2008; Dal Canto *et al*., 2012; Kim *et al*., 2017). However, it is a consensus that tPNf can be used as starting point when IVF or ICSI are used as a fertilization method (Cruz *et al*., 2013; Bodri *et al*., 2015).

When tPNf was used as a starting point, there were any observed differences in the cleavage stage (early and late) between the IVF and the ICSI techniques (Cruz *et al*., 2013; Bodri *et al*., 2015), although one of them reported a faster development in the blastocyst stage in IVF- fertilized embryos (Bodri *et al*., 2015). This could be explained because when IVF is performed the semen characteristics are superior to those in ICSI (Cruz *et al*., 2013; Bodri *et al*., 2015). Under this evidence, confounding factors should also be considered when both techniques are compared.

## OXYGEN CONCENTRATION AND UNINTERRUPTED CULTURE CONDITIONS

Oxygen concentration differs in the female reproductive tract, and it decreases from the fallopian tubes towards the uterus. The concentration ranges from 5-7% to 2% (Ng *et al*., 2018). Oxygen can influence embryo development. It is well documented that embryos cultured under lower oxygen concentration (5%) have more cleavage, good quality and more embryos are cryopreserved, which improved embryo implantation compared to those embryos cultured under higher oxygen concentration (20%) (Sciorio & Smith, 2019; Van Montfoort *et al*., 2020). Similar results were also found in the TL-system. A study reported a low quality of embryos, a lower number of utilized embryos and delays in s3 when the embryos were cultured in high-oxygen concentration (20%) (Kirkegaard *et al*., 2013b).

Additionally, in the TL-system, the embryos do not need to be removed from the incubator, this minimizes environmental disturbances caused by bench-top incubators. Interestingly, a study reported a higher live birth rate in embryos cultured in the TL-system, when compared to embryos cultured in high-quality K-systems, both using 5% O_2_ (Kalleas *et al*., 2020).

## COLLAPSE

Collapse is a separation of all or part of the trophectoderm (TE) cells from the zona pellucida (ZP) during blastocyst growth, the cells could be separated; when more than 50% form the inner side of the ZP (collapse or strong contraction) or less than 50% (weak contraction) (Marcos *et al*., 2015).

Blastocyst collapse is independent of the insemination technique (Sciorio *et al*. 2020a;b) and maternal age (Bodri *et al*., 2016; Gazzo *et al*., 2020). Whereas the relationship between collapse and embryo quality is arguable, because a study did not find a relation (Marcos *et al*., 2015), while two studies proposed a higher incidence of blastocyst collapse in poor quality embryos (Bodri *et al*., 2016; Sciorio *et al*., 2020b).

Embryos at the blastocyst stage could collapse at least one time. However, not all the blastocyst collapses (Marcos *et al*., 2015; Bodri *et al*., 2016; Viñals Gonzalez *et al*., 2018; Sciorio *et al*., 2020a;b). Retrospectives studies in TL showed that embryos that collapse during their development have lower implantation and pregnancy rates, especially if they were multiple when compared to blastocysts that did not display this event, although there are no relations between collapse duration and decreased implantation rates (Marcos *et al*., 2015; Bodri *et al*., 2016; Viñals Gonzalez *et al*., 2018; Gazzo *et al*., 2020; Sciorio *et al*., 2020a;b).

Embryo collapse and aneuploidy has also been investigated. It is reported that collapses were predominantly present in aneuploid embryos; moreover, embryos affected with monosomies have lower numbers of collapses when compared with trisomy or complex aneuploidies (Viñals Gonzalez *et al*., 2018). Even if an euploid embryo has collapses, it has a lower live-birth rate (Harton *et al*., 2016).

According to morphokinetic parameters, embryos without collapse had a slow development during early stages (t2,t3,t4 and tB) compared to those blastocyst which underwent blastocyst collapse (Marcos *et al*., 2015); while other studies reported that collapsed embryos took longer to reach the blastocyst stage (Gazzo *et al*., 2020), and a delayed development during the blastocyst stage (Bodri *et al*., 2016).

## RE-EXPANSION IN THAWED EMBRYOS

Freezing embryos is a common practice. It enables a single embryo transfer, genetics test or freeze all strategy for better endometrium preparation. During vitrification processes, embryos are dehydrated through the addition of cryoprotectants and, consequently, they shrunk. Immediately after warming, the embryos look collapsed, and need more time to recover their initial volume (re-expansion).

Blastocyst quality evaluation is difficult after the warming process. For that reason, post-warmed culture helps assess the vitrified/warmed blastocyst (Zhao *et al*., 2019). A study using the TL-system reported that re-expansion started as early as 10 minutes and can complete the re-expansion in 2 hours after warming (Ebner *et al*., 2017).

Embryos without re-expansion after the warming process have a significantly lower implantation rate when compared with the completely or partially re-expanded blastocyst (Desai *et al*., 2016; Coello *et al*., 2017; Ebner *et al*., 2017). In addition, after warming, the embryos can also have collapses, even more than once. However, it does not seem to affect implantation (Coello *et al*., 2017; Ebner *et al*., 2017). Shorter-duration re-expansion (from start to complete re-expansion) has been documented in those blastocysts that result in pregnancy in comparison to non-pregnant women (Ebner *et al*., 2017).

Furthermore, a predictive implantation model that evaluated morphology in post-warmed blastocyst has been proposed. The embryos were subdivided into four categories from A to D. If the maximum area values were >14,597mm^2^, the blastocysts were categorized as A or B, depending on whether the initial area values were >9,900mm^2^ or < 9,900mm^2^, respectively. Similarly, if the maximum area values were < 14,597mm^2^, the blastocysts were categorized as C or D, depending on the initial area. They found that implantation was 47.3%, 43.7%, 32.8 % and 14.2% for A to D, respectively (Coello *et al*., 2017).

## ATYPICAL DYNAMIC EMBRYO BEHAVIORS RELATED TO LOWER IMPLANTATION

Atypical dynamic embryo behaviors could happen during their early development, and are not maternal-age dependent (Athayde Wirka *et al*., 2014; Zhan *et al*., 2016; Yang *et al*., 2018). Embryos with anomalous divisions are more likely to arrest their development than embryos with normal divisions, especially during their first stages of development (Lagalla *et al*., 2017). Moreover, their embryo potential and implantation rates are adversely affected (Meseguer *et al*., 2011; Hlinka *et al*., 2012; Hur *et al*., 2018; Yang *et al*., 2018). These abnormal behaviors can only be visualized through continuous observation and subsequent time-lapse analysis.

Although, several atypical dynamic embryo behaviors have been described, two have shown to play a major role: Direct Cleavage (DC) and Reverse Cleavage (RC).

Direct Cleavage is defined as the second cell cycle being shorter than 5 hours, or more than 2 cells originated from a single cell division event. DC can occur during cleavage and their frequency is higher during the first cleaving periods (Fan *et al*., 2016); moreover, it could be present more than once (Zhan *et al*., 2016). It is noteworthy that DC in embryos, especially at earlier stages, strongly correlates with impaired blastocyst formation, implantation and clinical outcome (Rubio *et al*., 2012; Athayde Wirka *et al*., 2014; Fan *et al*., 2016; Zhan *et al*., 2016).

DC embryos are more often found during IVF cycles than ICSI cycles (Zhan *et al*., 2016), at higher percentages when testicular and epididymal sperm are used (Kahraman *et al*., 2020; Zhan *et al*., 2016). Additionally, DC embryos have 2.5 to 3.1 fold higher likelihood of a multinucleation incidence (Zhan *et al*., 2016).

Likewise, two retrospective studies suggest that DC embryos can undergo self-correction mechanisms while excluding some cells (with higher incidence of aneuploidies) during the compaction process. Therefore, DC embryos showed a similar euploid rate when compared to non-DC blastocysts on day 5 (Zhan *et al*., 2016)4. Nevertheless, this mechanism is significantly lower in older patients (>39 years) (Lagalla *et al*., 2017).

Reverse Cleavage is defined as either blastomeres rejoining after complete separation, or the blastomere fails to separate. It could occur during the cleavage stage, with major incidence during the third cycle of the mitotic division (5 to 8 cells) (Liu *et al*., 2014). The mean time of occurrence reported was 46.07h (Quera *et al*., 2014) and between 24 to 136h (Hickman *et al*., 2013).

In addition, RC affect the blastocyst stage development (Desai et al., 2018). Embryos with RC have significantly lower good-quality embryos on day 3 (Liu *et al*., 2014), and consequently a lower blastocyst formation rate (Yang *et al*., 2018). Furthermore, RC embryos implanted less than their counterparts (Liu *et al*., 2014), especially if two or more abnormal cleavages were present in a single embryo, increasing the likelihood of the embryo being aneuploid (Desai *et al*., 2018).

## PLOIDY

A meta-analysis concluded that Time-lapse embryo monitoring may help predict the ploidy of embryos (Swain, 2013), even though other studies proposed that morphokinetics are not enough (Reignier *et al*., 2018); but it provides valuable information for embryo selection (Zaninovic *et al*., 2017).

Some studies did not find differences during the cleavage and blastocyst stages of embryo development between euploids and aneuploids (Stevens *et al*., 2012; Semeniuk *et al*., 2013; Yang *et al*., 2014) (Table 3). Conversely, others have reported a development delay in aneuploid embryos during cleavage and blastocyst stages (Table 3) (Davies *et al*., 2012; Campbell *et al*., 2013; Vera-Rodriguez *et al*., 2015; Chawla *et al*., 2015; Mumusoglu *et al*., 2017; Zhang *et al*., 2017; Huang *et al*., 2019).

**Table 3.** Summary of the studies that combine Time lapse embryo culture and PGT-A analysis.

Item	Reference	Type of study	Embryos (n)	Patients (n)	Embryo culture	Day transfer	Morphokinetic parameters		Statistically significant variables
							significant difference	no significant difference	
Euploid vs. aneuploid	Amir *et al*., 2019	cohort study	270	NA	Blastocyst stage	FISH	x		t4,tSB, tPNf
	Basile *et al*., 2014	Retrospective	504	125	Blastocyst stage	aCGH	x		t5-t2, cc3
	Bayram *et al*., 2012	Prospective	122	17	Cleavage stage	FISH	x		s3
	Campbell *et al*., 2013	Retrospective	195	25	Blastocyst stage	aCGH	x		tSC,tSB, tB
	Chavez *et al*., 2012	Cohort studies	53	NA	Cleavage stage	aCGH	x		from t1 to t4
	Chawla *et al*., 2015	Retrospective	496	132	Blastocyst stage	aCGH	x		tPNf, t2, t5,cc2,cc3, t5-t2
	Davies *et al*., 2012	Retrospective	62	NA	NA	aCGH	x		t2,t3,s2
	Del Carmen Nogales *et al*., 2017	Retrospective	485	112	Blastocyst stage	aCGH	x		t3, t5, cc2, cc3, s2, t5-t2
	Hong *et al*., 2013	Prospective	307	24	Blastocyst stage	qPCR	x		tEB and from t5 to tSB
Euploid vs. aneuploid	Huang *et al*., 2019	Retrospective	188	34	Cleavage stage	aCGH	x		tBE
	Kahraman *et al*., 2020	Retrospective	741	279	Blastocyst stage	aCGH	x		t2,t3,t4,t5,t6,t7,t8,t9, tM, tSB, tB
	Kramer *et al*., 2014	Retrospective	149	25	Blastocyst stage	aCGH		x	
	Melzer et al., 2013	Prospective							tM
	Minasi et al., 2016	Retrospective	928	454	Blastocyst stage	aCGH	x		t4,s2,tB, tEB, tHed
	Mumusoglu *et al*., 2017	Retrospective	415	103	Blastocyst stage	aCGH	x		t9,tM,tSB,tB,tEB
	Patel *et al*., 2016	Retrospective	167	26	Blastocyst stage	aCGH		x	
	Rienzi *et al*., 2015	cohort study	455	138	Blastocyst stage	aCGH		x	
	Semeniuk *et al.*, 2013	retrospective	76	NA	Blastocyst stage	aCGH		x	
Euploid vs. aneuploid	Stevens *et al*., 2012	Cohort studies	53	35	Blastocyst stage	qPCR		x	
	Vera-Rodriguez *et al*., 2015	Prospective	85	NA	Cleavage stage	aCGH	x		tPNf, t2 , s2
	Yang *et al*., 2014	prospective	498	138	Blastocyst stage	aCGH		x	
	Zhang *et al*., 2017	Retrospective	256	75	Blastocyst stage	aCGH		x	
Male vs. female	Bodri *et al*., 2016	Retrospective	291	NA	Blastocyst stage	NA	X		t8,tM, tB
	Bronet *et al*., 2015	Retrospective	327	93	Blastocyst stage	aCGH	X		s2, tM
	Huang & Jin, 2017	Retrospective	174	134	NA	NA	X		t3,t4,cc2
	Huang *et al*., 2019	Retrospective	308	228	Cleavage stage	NA	X		t3,t4,t5, cc2
Male vs. female	Melzer *et al*., 2013	Prospective	213	20	Blastocyst stage	aCGH	X		t4
	Serdarogullari *et al*., 2014	Retrospective	177	139	NA	NA		x	
	Zeyad *et al*., 2018	cohort study	416	120	Blastocyst stage	FISH	X		tPN, tPNf

Note: NA: not available; d2/3/5: day 2/3/5; FISH: fluorescence in situ hybridization; aCGH: array comparative genomic hybridization; qPCR: quantitative polymerase chain reaction; other abbreviations in Table 1.

Similarly, a prospective study reported that chromosomally normal embryos display strict and tightly clustered cell cycle parameters up to the 4-cell stage (Chavez *et al*., 2012). Other studies found shorter stages in euploid embryos than aneuploid embryos: in s3 (Bayram *et al*., 2012), a shorter compaction (Melzer *et al*., 2013) and early cavitation from first cytokinesis and from t5 (Hong *et al*., 2013), a shorter cleavage s2 and t4 as well during the blastocyst stage tB, tEB and tHed (Minasi *et al*., 2016), a faster development from t2 to blastocyst stage; however, these differences are not present in severe forms of male infertility (less than 1x10^6^ spermatozoa /ml) (Kahraman *et al*., 2020).

In addition, a retrospective study reported that embryos with trisomy showed very similar kinetics to those of normal embryos, whereas embryos with monosomies fall between complex and trisomy embryos (Del Carmen Nogales *et al*., 2017). Furthermore, embryos with complex chromosomal abnormalities have the shortest division times (t3, t5, cc2, cc3, s2 and t5-t2), which is strongly associated with t3 and t5-t2 (time interval between the 2- and the 5-cell stage) (Del Carmen Nogales *et al*., 2017). Moreover, a study reported that unbalanced chromosomal translocation embryos showed a delay of t4, tSB and s2, and embryos with balanced translocation did not; even a delay in tPNf was seen in embryos with nonviable unbalanced chromosomal translocation, when compared to potentially viable embryos (Amir *et al*., 2019).

Likewise, there are models proposed to identify embryos more likely to be euploid, based on variables. Some studies have identified that t5-t2, cc3, tSB and tB are good predictor variables (Basile *et al*., 2014; Campbell *et al*., 2013); others have been more specific, a cc3 (t5-t3) >10.00h and t5-t2 > 20.00h (Chawla *et al*., 2015), and blastocyst initiation (tSB>96.2h); progression to expanded blastocysts (tSB>166h) and tEB- tSB >13h in aneuploid embryos (Desai *et al*., 2018) are morphokinetic parameters associated with aneuploidy.

On the other hand, other studies demonstrated that the models described above were unable to discriminate between euploid and aneuploid embryos (Kramer *et al*., 2014; Rienzi *et al*., 2015; Patel *et al*., 2016; Zhang *et al*., 2017).

### Sex

A single study with 177 embryos reported that female embryos showed earlier cavitation than male embryos, but it did not reach statistical significance (Serdarogullari *et al*., 2014). However, studies with aCGH analysis found a faster development in male embryos. A single study found that male embryos tend towards a faster progression to t4 than female embryos, with 213 embryos included (Melzer *et al*., 2013); while a study with 327 embryos also found a faster development in male embryos. Hence, they proposed an algorithm based on s2 and tM that permits identifying embryos with higher probability of being female (Bronet *et al*., 2015). By contrast, a study evaluated 416 embryos through the FISH technique, and they reported that tPN is significantly faster in female than male embryos; whereas tPNf was significantly faster in male embryos; and the blastulation rate was significantly higher in female embryos (Zeyad *et al*., 2018).

As described above, a retrospective study based on 81 live births reported a significantly slower development for tB in male embryos than in female embryos (Bodri *et al*., 2016). Meanwhile, retrospectives studies reported t3, t4 and cc2 earlier in male embryos than those of female embryos (Huang & Jin, 2017); as well as t3,t4,t5 and cc2 (Huang *et al*., 2019). However, only t3 (<14h) was correlated with live birth sex (Huang *et al*., 2019).

## CONCLUSIONS

Time Lapse provides valuable information and enormous potential to enhance our understanding of embryo development. Considerable knowledge has been accumulated and describes the morphokinetic dynamics during different stages in human embryos. It has been demonstrated that some patient characteristics and culture conditions modified this development pattern. There is a consensus that embryos generated from advanced maternal age have a slow development. Moreover, the current available data about smoking and high-oxygen concentration cultures are scarce, but it also demonstrated a slow development in both groups. On the contrary, any difference in morphokinetics has been described when IVF or ICSI was used as the fertilization method. Similar observations were found when the embryos were culture in single or sequential media.

On the other hand, the limited number of studies, the considerable differences in the study designs and patients' characteristics like BMI, PCOS, endometriosis, ploidy and gender made it difficult to draw a conclusion. Given this inconsistency and lack of evidence, more prospective studies and further randomized clinical trials are needed.

The association between morphokinetics and ART outcome has important implications in clinical results; where two morphokinetic events have the potential to predict embryo implantation: blastocyst collapse-re-expansion and direct-reverse cleavage seems to have a strong impact on clinical outcomes.
